# Features and Advantages of Supercritical CO_2_ Extraction of Sea Cucumber *Cucumaria frondosa japonica* Semper, 1868

**DOI:** 10.3390/molecules25184088

**Published:** 2020-09-07

**Authors:** Alexander Zakharenko, Denis Romanchenko, Pham Duc Thinh, Konstantin Pikula, Cao Thi Thuy Hang, Wenpeng Yuan, Xuekui Xia, Vladimir Chaika, Valery Chernyshev, Svetlana Zakharenko, Mayya Razgonova, Gyuhwa Chung, Kirill Golokhvast

**Affiliations:** 1 School of Biomedicine, Department of Pharmacy and Pharmacology, Far Eastern Federal University, Sukhanova 8, 690950 Vladivostok, Russia; dromanch@mail.ru (D.R.); k.pikula@mail.ru (K.P.); chaika.vv@dvfu.ru (V.C.); chernyshev.vv@dvfu.ru (V.C.); droopy@mail.ru (K.G.); 2 N.I. Vavilov All-Russian Institute of Plant Genetic Resources, 190000 Sankt-Peterburg, Russia; rarf3340@yandex.ru (S.Z.); Razgonova.mp@dvfu.ru (M.R.); 3 NhaTrang Institute of Technology Research and Application, VAST, Nhatrang, Nha Trang City 57000, Vietnam; ducthinh.nitra@gmail.com (P.D.T.); caohang.nitra@gmail.com (C.T.T.H.); 4 Heze Branch, Qilu University of Technology (Shandong Academy of Sciences), Biological Engineering Technology Innovation Center of Shandong Province China, Heze 274008, China; yuanwp@sdas.org; 5 Key Laboratory for Biosensor of Shandong Province, Biology Institute, Jinan 250013, China; xiaxk@sdas.org; 6 Department of Biotechnology, Chonnam National University, Yeosu 59626, Korea; chung@chonnam.ac.kr

**Keywords:** sea cucumber, supercritical extraction, *Cucumaria frondosa japonica*, triterpene glycosides, saponins, carotenoids, tandem mass spectrometry

## Abstract

Extraction process of *Cucumaria frondosa japonica* Semper, 1868, which are subspecies of *Cucumaria frondosa* (Gunnerus, 1767), were studied. It was shown that supercritical carbon dioxide extraction of holothuria was more effective than conventional solvent extraction. Step-by-step extraction with carbon dioxide followed by supercritical extraction with the addition of a co-solvent of ethanol can almost double the yields of extracts of triterpene glycosides, styrenes and carotenoids. Moreover, the fraction of triterpene glycosides practically does not contain colored impurities, in contrast to traditional ethanol extraction. The obtained extracts by HPLC in combination with tandem mass spectrometry (HPLC-MS/MS) identified 15 triterpene glycosides, 18 styrene compounds and 14 carotenoids. Supercritical extraction made it possible to obtain extracts with yields superior to conventional hexane and alcohol extracts. Moreover, such an approach with the use of supercritical fluid extraction (SFE) and subsequent profiling of metabolites can help with the study of holothuria species that are not as well studied.

## 1. Introduction

Oceans are the least studied part of the Earth. Every year, dozens of new compounds are isolated and described from marine hydrobionts around the world. Many of them have different biological activities and many have been used in drug development. Secondary metabolites of the representatives of the class of echinoderms are of great practical and fundamental interest. New secondary metabolites isolated from echinoderms are discovered annually. Many articles have been published describing the features of the mechanisms of biological action of previously discovered compounds. Of particular interest are compounds isolated from holothurians and sea urchins [1,2,3].

Sea cucumbers are marine invertebrates that belong to Echinodermata, Holothuroidea. Class Holothuroidea are widely found in the benthic area and deep seas worldwide. Components of sea cucumbers possess different biological activities such as anticancer, antiviral, antioxidant and wound healing. Recently, much attention has been paid to isolation, to determine the structure and study of the properties of sulfated polysaccharides from sea cucumbers [4].

Supercritical fluid solvents represent interesting alternatives for producing high-quality natural food products without toxic residues [5]. The introduction of supercritical fluid extraction (SFE) has led to a novel technology that is being continually developed [6,7]. High-pressure SFE can be used to produce natural thermolabile compounds, leaving no organic solvent residues in food products, which are commonly observed with conventional extraction methods using methanol and hexane [8].

## 2. Results

### 2.1. Sterol Compositions Identified in Supercritical CO_2_ Extract

Profiling of styrenes from the whole-body extract of the sea cucumber using the LC-MS approach allowed numerous new as well as previously isolated triterpene glycosides to be characterized. The total yield of sterol fraction was 114 mg that corresponded to 190 mg/100 g. To identify sterol components, this fraction was hydrolyzed. Alkaline hydrolysis was performed in 2 mL ethanol and 0.15 mL oversaturated KOH solution at 85 °C for 30 min. Then to the reaction mixture, the upper layer was collected and analyzed with HPLC ESI MS-MS. The main sterol components were diatomsterol and cholestanol 20.2 and 13.2% (Table 1).

### 2.2. Carotenoids Identified in Supercritical CO_2_ Extract

During the first step, SFE fatty fraction was isolated. Total yield was 1.1 g. Fourteen carotenoids were identified with a total mass of 71.1 mg (Table 2). The major component of carotenoid fraction was Cucumariaxanthin yielding 33.6 mg, that corresponded to 56.1 mg per 100 g of dry weight. The second main carotenoid was Canthaxanthin, 25.4 mg/100 g. The third main component was Cucumariaxanthin B. Lutein was detected in the smallest amount 0.5 mg/100 g.

### 2.3. Triterpene Glycosides Identified in Supercritical CO_2_ Extract

In the second step, SFE extracts were analyzed by HPLC in combination with tandem mass spectrometry (HPLC ESI MS/MS). A total of 15 different triterpene glycosides were identified (Table 3).

**Figure 1:**
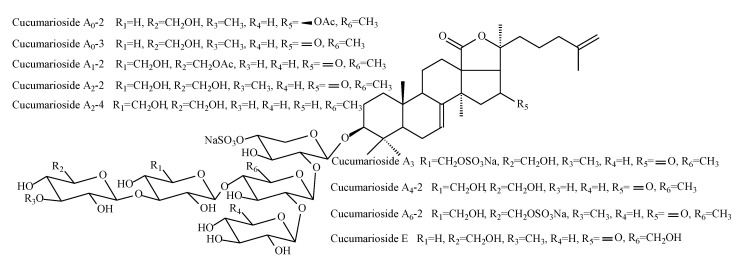
Structure of triterpene glycosides from the supercritical extract of *C. frondosa japonica*.

**Figure 2:**
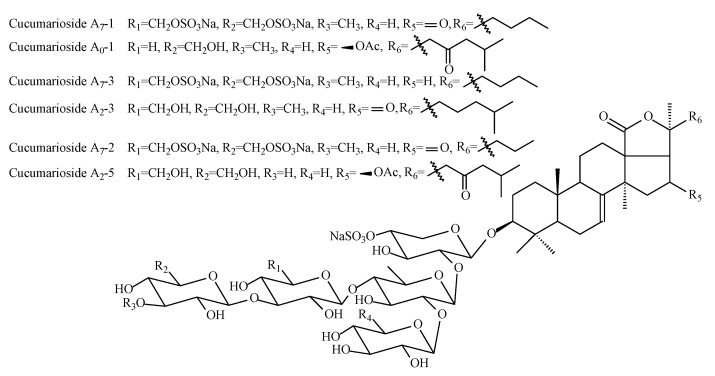
Structure of triterpene glycosides from the supercritical extract of *C*. *frondosa japonica*.

All of the detected triterpene glycosides were found earlier in this type of holothuria, but this is the first study when it was possible to detect all of these glycosides at the beginning of one complex experiment. The structural formulas of the detected glycosides are shown in Figure 1 and Figure 2.

**Figure 3:**
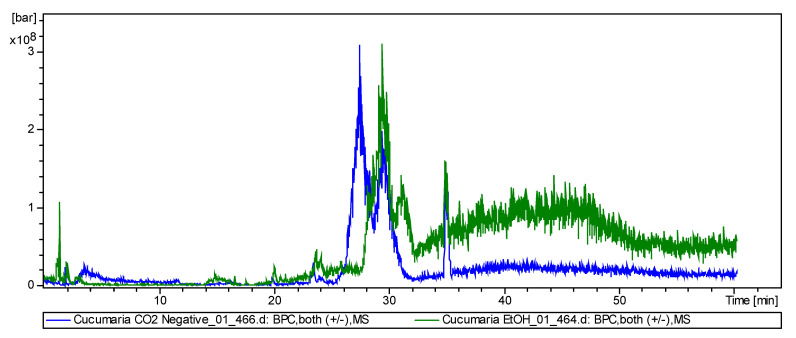
Liquid chromatography–electrospray mass spectrometry (LC-ESI MS) total compounds chromatogram of detected triterpene glycosides in negative ion mode (sulfated, disulfated and non-sulfated glycosides were detected as [M−Na]^−^, [M−2Na]^2−^ and [M−H]^−^ ions) in supercritical extract of *C. frondosa japonica*.

Total compounds chromatogram of detected triterpene glycosides in negative ion mode shown in Figure 3. The figure shows that the total fraction without preliminary separation has poorly resolved peaks, and although the full qualitative composition can be determined due to the mass spectrometer detector, this does not allow quantitative analysis.

## 3. Discussion

*Cucumaria frondosa japonica* Semper, 1868 is a subspecies of Cucumaria frondosa (Gunnerus, 1767). *Cucumaria frondosa japonica*’s habitat is in the Pacific Ocean but does not occur in the Atlantic Ocean. These two species have very similar metabolites but are slightly modified. Both of these are commercial species of holothuria, are widely used in food and are a source of concentrates for the pharmaceutical industry [9,10]. All this has led to the fact that over the past 40 years, the biochemical composition of this species has been fairly well studied. One of the first works on the biochemistry of this holothurian dates back to 1980. The triterpene glycoside of these holothurians is most well studied, since they are promising substances for medicine [11,12,13]. Use of supercritical extraction allowed us to increase the yield of the total fraction with triterpene glycosides and for the first time in one complex experiment we showed all 15 triterpene glycosides present in *Cucumaria frondosa japonica*. For simultaneous qualitative characterization of the present triterpene glycosides, we used the metabolite profiling approach used earlier by other groups for the analysis of triterpene glycosides [14,15]. This approach allows reliable identification of known molecules, even if they are not well separated during HPLC.

The first stage of extraction was carried out at a pressure of 2500 psi, which allows the extraction of almost only hydrophobic nonpolar and weakly polar molecules, such as carotenoids, fats and sterols. This was used to analyze the carotenoids and sterols contained in *Cucumaria frondosa japonica*. Supercritical extraction made it possible to obtain extracts with yields superior to conventional hexane and alcohol extracts. Moreover, such an approach with the use of SFE and subsequent profiling of metabolites can help with the study of holothuria species that are not as well studied as *Cucumaria frondosa japonica*. This approach helps to better study the carotenoid composition and minor components of other types of Echinodermata. Supercritical extraction is a very promising approach and since it avoids unnecessary oxidation of substances, prevents unpleasant odors, improves product quality and yield, and is more profitable to operate.

## 4. Materials and Methods

### 4.1. Sea Cucumber

The live specimens of sea cucumber *Cucumaria frondosa japonica* Semper, 1868, were collected in Peter the Great Gulf (Russia, Sea of Japan) in 2019. The body wall of the sea cucumbers, total weight 1450 g, was dissected free of intestine and detritus with scissors, cut into small pieces (about 2 cm long), and stored in a polyethylene bag at −70 °C until used. Later, sea cucumbers with the total weight of 1450 g, were freeze-dried to 120 g, then the sample was separated into two equal parts, for ethanol extraction and supercritical fluid extraction (SFE).

### 4.2. Chemicals

Acetonitrile HPLC-grade was purchased from (PANREAC, Barcelona, Spain), formic acid and KOH were purchased from (Sigma-Aldrich, St. Louis, MO, USA). Ethanol was purchased from (LLC ORT “Khimreaktivy”, Yekaterinburg, Russia). For all experiments we used deionized water (Siemens Ultra-Clear TWF Water Purification Systems, Günzburg, Germany).

### 4.3. Supercritical Fluid Extraction

Frozen sea cucumbers were freeze dried and minced. Dry weight was 120 g. Then 60 g was extracted in supercritical CO_2_ pressure extraction apparatus (Thar SFC 500, San Diego, CA, USA). Extraction was carried out in two steps, first to get carotenoids and sterols fraction with CO_2_ under the pressure of 2500 psi, at a temperature of 60 °C, and flow rate was 50 mL/min for liquid CO_2_. flow of 20 g/min. Total yield was 1.1 g or 1.8% from dry weight. The second step was with a flow rate 50 mL/min for liquid CO2 and 1.00 mL/min for EtOH under the pressure of 7000 psi, at a temperature of 60 °C, and a flow of 20 g/min. Yield was 3.4 g and approximately 1.1 g was triterpene glycosides. Another 60 g of dried sea cucumbers were used for usual ethanol extraction.

### 4.4. Ethanol Extraction

The second part of freeze-dried body with the weight of 60 g was extracted with ethanol. The extraction was carried out at a ratio of 1:2 in a dark container at a temperature of 40 °C, with constant alternation on a stirrer for 3 h. Then the extract was filtered off and another 60 mL of ethanol was poured into the precipitate for re-extraction within 3 h. The precipitates were combined and concentrated in a rotary evaporator under vacuum at a temperature of 40 °C and then evaporated under vacuum until a constant mass was yielded. Total yield was 2.7 g.

### 4.5. High-Performance Liquid Chromatography-Electrospray Ionization Tandem Mass Spectrometry

The samples and calibration solutions were filtered (0.22 μm Whatman filters) and injected into the chromatographic system (loop volume 20 μL). Reverse-phase HPLC was performed with Shimadzu LC-20 liquid chromatography (Kanda-Nishikicho 1-chrome, Shimadzu, Chiyoda-ku, Tokyo, Japan) equipped with column oven CTO-20A (Kanda-Nishikicho 1-chrome, Shimadzu, Chiyoda-ku, Tokyo, Japan) and UV-VIS detector SPD-20A (Kanda-Nishikicho 1-chrome, Shimadzu, Chiyoda-ku, Tokyo, Japan). The analytical column used was an ZORBAX Eclipse XDB C18 (150 × 4.6 mm, Pore Size: 80, Particle Size: 5.0 µ, Carbon Load: 1%, Reversed Phase C18 (Octadecyl), manufacturer: Agilent Technologies Inc., Wilmington, DE, USA) at 30 °C and the total flow rate 0.22 mL min^−1^. The gradient elution program with two mobile phases (A, deionized water; B, acetonitrile with formic acid 0.1% *v*/*v*) was as follows 0 min 0% B, 25 min 100% B, 60 min 100% B. To study the fraction containing triterpene glycosides, the liquor was previously concentrated, and purified by low pressure hydrophobic chromatography on a Polychrome-I (Polytetrafluoroethylene granules).

ESI-MS and ESI-MSn spectra were recorded using an amaZon SL ion trap (BRUKER DALTONIKS, Bremen, Germany) equipped with an electrospray ion source. ESI MS ionization parameters were optimized as follows: a capillary voltage of 4500 V, end plate bend voltage: 1500V, collision energy: 60 eV, nebulization with nitrogen at 29 psi, dry gas flow of 10 L/min at a temperature of 160 °C. The mass spectra were recorded within *m*/*z* mass range of 500–2000 in negative and positive ion mode. Product ion mass spectra were recorded in auto MS/MS mode. Identification of compounds was carried out according to the Bruker library and literature data [11,16,17].

## 5. Conclusions

It was shown that supercritical carbon dioxide extraction of holothuria *Cucumaria frondosa japonica* Semper, 1868 was more effective than conventional solvent extraction. Supercritical extraction is a very promising approach since it avoids unnecessary oxidation of substances, prevents unpleasant odors, improves product quality and yield, and is more profitable to operate. Step-by-step extraction with carbon dioxide followed by supercritical extraction with the addition of a co-solvent of ethanol can almost double the yields of extracts of triterpene glycosides, styrenes and carotenoids. Moreover, the fraction of triterpene glycosides practically does not contain colored impurities, in contrast to traditional ethanol extraction. The obtained extracts by HPLC in combination with tandem mass spectrometry (HPLC-MS/MS) identified 15 triterpene glycosides, 18 styrene compounds and 14 carotenoids. Separation of the triterpene glycoside fraction into sub-fractions by hydrophobic chromatography on a Polychrome-I carrier made it possible to additionally identify 3 minor triterpene glycosides cucumarioside A_7-1_, cucumarioside A_7-2_ and cucumarioside E. We managed to isolate all triterpene glycosides, including minor ones, but no new triterpene glycosides were found. This publication is primarily of methodological and technological value. These data will be useful for organizing production facilities for the commercial isolation of valuable products from mountainuria and for studying the composition of triterpene glycosides of not so widespread and well-studied species in order to discover new triterpene glycosides.

## Figures and Tables

**Table 1:** Sterol components identified from the supercritical extract of *C. frondosa japonica* Semper, 1868.

№	Sterol	Molecular Formula	[M+H]^+^ (*m*/*z*)	MS2 (*m*/*z*)	Yield, %
1	24-nor-5α-cholesta-7,22-dien-3β-ol (Asterosterol)	C_26_H_42_O	371.32	303.26; 275.23; 235.20	1.34
2	24-nor-5α-cholesta-22-en-3β-ol (24-nordehydrocholestanol)	C_26_H_44_O	373.34	305.28; 277.25; 237.21	1.48
3	5α-cholesta-7,22-en-3β-ol	C_27_H_44_O	385.34	329.28; 303.26; 235.20; 167.14; 143.14	9.26
4	5α-cholest-22-en-3β-ol (trans-22-dehydrocholestanol)	C_27_H_46_O	387.35	331.29; 305.28; 237.21; 169.15; 143.14	6.06
5	5α-cholest-7-en-3β-ol (lathosterol)	C_27_H_46_O	387.35	331.29; 303.26; 235.20; 167.14; 143.14;	3.24
6	5α-cholestan-3β-ol (cholestanol)	C_27_H_48_O	389.37	333.31; 305.28; 237.21; 169.15; 143.14	13.20
7	24-methyl-5α-cholesta-trien-3β-ol	C_28_H_44_O	397.34	327.26; 299.23; 231.17; 165.12; 141.12	1.94
8	24-methyl-5α-cholesta-7,22-dien-3β-ol (diatomsterol)	C_28_H_46_O	399.35	329.27; 303.26; 235.20; 167.14; 141.12	20.22
9	24-methylen-5α-cholest-7-en-3β-ol	C_28_H_46_O	399.35	331.29; 235.20; 167.14; 141.12	2.40
10	24-methyl-5α-cholest-7-en-3β-ol	C_28_H_48_O	401.37	331.29; 235.20; 167.14; 141.12	1.88
11	24-methyl-5α-cholest-22-en-3β-ol (diatomstanol)	C_28_H_48_O	401.37	331.29; 237.21; 169.15; 143.14	2.78
12	24-methylen-5α-cholestan-3β-ol	C_28_H_48_O	401.37	333.31; 237.21; 169.15; 143.14	9.28
13	24-methyl-5α-cholestan-3β-ol (campestanol)	C_28_H_50_O	403.39	333.31; 237.21; 169.15; 143.14	3.64
14	24-ethyl-5α-cholesta-trien-3β-ol (Provitamin D6)	C_29_H_46_O	411.36	327.26; 233.18; 165.12; 140.12	0.64
15	24-ethyl-5α-cholest-7,22-dien-3β-ol (Spinasterol)	C_29_H_48_O	413.38	329.28; 235.20; 167.14; 141.12	4.44
16	24-ethyl-5α-cholest-7-en-3β-ol (Scottenol)	C_29_H_50_O	415.39	331.29; 235.20; 167.14; 141.12	9.06
17	24-ethyl-5α-cholest-22-en-3β-ol (stigmast-22E-en-3β-ol)	C_29_H_50_O	415.39	331.29; 237.21; 169.15; 143.14	2.66
18	24-ethyl-5α-cholestan-3β-ol (sitostanol)	C_29_H_52_O	417.40	333.31; 237.21; 169.15; 143.14	7.82

**Table 2:** Components identified from the supercritical extract of *C. frondosa japonica* Semper, 1868.

№	Identity	Molecular Formula	MS (*m*/*z*)	MS2 (*m*/*z*)	Yield, (mg/100 g)
1	β-Carotene	C_40_H_56_	537.5	445.4; 379.4; 346.3; 308.3; 268.3; 224.2; 203.2; 178.2; 133.2; 119.1; 107.1; 95.1	4.2
2	β-Echinenone	C_40_H_54_O	551.5	347.3; 265.3; 209.2; 203.2;157.2; 133.1; 119.1; 95.1; 81.1; 69.1	0.9
3	Canthaxanthin	C_40_H_5__2_O_2_	565.5	548.5; 413.3; 404.0; 363.3; 307.3; 231.2; 215.2; 203.2; 145.2; 133.2; 95.2; 69.1	25.4
4	(3R)-, (3S)-phoenicoxanthin	C_40_H_5__2_O_3_	581.5	565.5; 562.5; 488.4; 157.0; 119.0; 105.0; 91.0; 55.0	1.6
5	Lutein	C_40_H_56_O_2_	568.5	550.5; 476.4; 430.4; 367.0; 336.3; 323.3; 175.2; 145.2; 133.2; 107.1; 95.2	0.5
6	Diatoxanthin	C_40_H_54_O_2_	567.5	551.5; 533.5; 459.4; 413.4; 329.3; 263.3; 217.2; 199.2; 175.2; 133.2; 109.2	1.8
7	Alloxanthin	C_40_H_5__2_O_2_	565.5	547.5; 491.4; 465.4; 411.3; 393.0; 249.2; 209.2; 199.2; 173.2; 157.2; 119.1; 109.2; 81.1	2.3
8	Pectenolone	C_40_H_5__2_O_3_	581.5	563.5; 315.3; 27.2; 217.2; 199.2; 173.2; 147.2; 119.1; 107.1	2.2
9	(3S,3′S)-7,8-Didehydroastaxanthin	C_40_H_50_O_4_	595.5	577.5; 565.5; 441.6; 425.4; 375.4; 359.4; 165.2	1.7
10	Fucoxanthin	C_42_H_58_O_6_	659.5	641.5; 623.5; 599.5; 581.5; 567.4; 549.4; 489.4; 433.3; 355.3; 239.2; 149.2; 109.2	1.4
11	Fucoxanthinol	C_40_H_56_O_5_	617.5	598.3; 447.4; 285.2; 233.2; 143.0; 109.2; 91.0; 43.0	1.6
12	Cucumariaxanthin A	C_40_H_56_O_2_	568.5	550.2; 476.4; 462.3	56.1
13	Cucumariaxanthin B	C_40_H_58_O_2_	571.1	552.4; 478.1; 464.3	13.7
14	Cucumariaxanthin C	C_40_H_60_O_2_	573.6	556.2; 536.3; 480.5; 466.2	5.1

**Table 3:** Triterpene glycosides identified from the supercritical extract of C. *frondosa japonica*.

№	Identity and Retention Time *	Molecular Formula	Adducts	MS (*m*/*z*)	MS2 (*m*/*z*)	MS3 (*m*/*z*)
1	cucumarioside A_0_-1 27.6 min	C_60_H_93_O_30_SNa	[M_Na_–Na]^−^	1325.55	1193.50; 797.20	1017.44; 885.39; 739.34; 665.16; 489.09; 357.05; 211.00
2	**cucumarioside A_0_-2** **30.3 min**	C_60_H_93_O_29_SNa	[M_Na_–Na]^−^	1309.55	1177.51; 797.20	1001.44; 869.40; 723.34; 665.16; 489.09; 375.05; 211.00
3	**cucumarioside A_0_-3** **30.8 min**	C_58_H_89_O_28_SNa	[M_Na_–Na]^−^	1265.53	1133.48; 797.20	957.42; 825.37; 679.32; 665.16; 489.09; 375.05; 211.00
4	cucumarioside A_1_-2 28.4 min	C_60_H_91_O_30_SNa	[M_Na_–Na]^−^	1323.53	1191.49; 855.21	987.43; 825.37; 723.17; 519.10; 357.05; 211.00
5	cucumarioside A_2_-2 28.6 min	C_59_H_91_O_29_SNa	[M_Na_–Na]^−^	1296.41	1163.49;	987.43; 827.21; 825.37; 695.17; 679.32; 519.10; 357.05; 211.00
6	cucumarioside A_2_-3 28.5 min	C_59_H_93_O_29_SNa	[M_Na_–Na]^−^	1297.55	1165.51; 827.21	989.44; 827.39; 695.17; 681.33; 519.10; 357.05; 211.00
7	cucumarioside A_2_-4 29.0 min	C_58_H_91_O_28_SNa	[M_Na_–Na]^−^	1267.54	1135.50;	974.45; 813.20; 811.39; 681.15; 665.33; 519.10; 357.05; 211.00
8	cucumarioside A_2_-5 28.5 min	C_60_H_93_O_31_SNa	[M_Na_–Na]^−^	1341.54	1209.50; 813.20	1047.45; 885.39; 739.34; 681.15; 519.10; 357.05; 211.00
9	cucumarioside A_3_ 29.4 min	C_59_H_90_O_32_S_2_Na_2_	[M_Na2_–Na]^−^	1397.48	1265.43; 929.15	1089.36; 825.37; 797.11; 679.32; 621.04; 357.05; 211.00
10	cucumarioside A_4_-2 29.6 min	C_58_H_89_O_29_SNa	[M_Na_–Na]^−^	1281.52	1149.48; 813.20	987.43; 825.37; 681.15; 519.10; 357.05; 211.00
11	cucumarioside A_6_-2 29.2 min	C_59_H_90_O_32_S_2_Na_2_	[M_Na2_–Na]^−^	1397.48	1265.43; 929.15	987.43; 825.37; 797.11; 679.32; 519.10; 357.05; 211.00
12	**cucumarioside A_7_-1** **27.3 min**	C_57_H_87_O_35_S_3_Na_3_	[M_Na3_–Na]^−^	1473.40	1341.35; 1031.09	1063.35; 899.05; 799.35; 659.19; 653.30; 621.04; 357.05; 211.00
13	**cucumarioside A_7_-2** **26.9 min**	C_56_H_85_O_35_S_3_Na_3_	[M_Na3_–Na]^−^	1459.38	1327.34; 1031.09	1049.33; 899.05; 785.34; 639.28; 621.04; 357.05; 211.00
14	cucumarioside A_7_-3 27.1 min	C_57_H_89_O_34_S_3_Na_3_	[M_Na3_–Na]^−^	1459.42	1327.38; 1031.09	1049.37; 899.05; 785.38; 639.32; 621.04; 357.05, 211.00
15	**cucumarioside E** **34.5 min**	C_58_H_89_O_29_SNa	[M_Na_–Na]^−^	1281.52	1149.51; 813.22;	1105.51; 973.42; 841.41; 679.32; 637.19; 505.2
			[M_Na_+Na]^+^	1327.48	1207.51	1075.42; 899.29

*—Bold letters used for those triterpene glycosides that were detected only in SFE fraction.
